# Herpes Zoster Ophthalmicus Complicated by Optic Neuritis: Diagnostic and Therapeutic Challenges

**DOI:** 10.7759/cureus.108343

**Published:** 2026-05-06

**Authors:** Narapa Reddy Meghana, Gayatri Sundareswaran, Venkat Meghana Bhimanadham, Narayanan Balakrishnan

**Affiliations:** 1 Department of Ophthalmology, Sri Ramachandra Institute of Higher Education and Research, Chennai, IND

**Keywords:** acyclovir, herpes zoster ophthalmicus (hzo), intravenous methyl prednisolone, neuroimaging, optic neuritis

## Abstract

Herpes zoster ophthalmicus (HZO) is caused by reactivation of the varicella-zoster virus (VZV) in the ophthalmic branch of the trigeminal nerve and can result in a range of ocular complications. One of the rare but serious sequelae of HZO is optic neuritis, which can lead to significant visual impairment within a few days without timely management. We present a case of an immunocompetent adolescent female who developed right-sided retrobulbar optic neuritis following an episode of HZO. Her condition was confirmed with magnetic resonance imaging (MRI), which showed optic nerve involvement. Management included intravenous and oral antivirals, along with high-dose corticosteroids, resulting in partial visual recovery.

This case underscores the need for heightened clinical suspicion for optic neuritis in patients presenting with HZO, even among young immunocompetent individuals. Prompt and aggressive antiviral and anti-inflammatory treatment, coupled with multidisciplinary management, is crucial for optimal visual outcomes.

## Introduction

Varicella-zoster virus (VZV) reactivation involving the ophthalmic branch of the trigeminal nerve is referred to as herpes zoster ophthalmicus (HZO). HZO accounts for approximately 8%-20% of all herpes zoster cases and presents with a painful vesicular rash in the distribution of the ophthalmic nerve [1]. While common ocular complications include conjunctivitis, keratitis, anterior uveitis, and secondary glaucoma, involvement of the posterior segment and optic nerve is less frequently observed. Optic neuritis following HZO is a rare complication, with an incidence of 0.4% of all HZO cases [2]. The pathophysiology of optic neuritis in HZO is multifactorial, including direct invasion of the virus into the optic nerve and ischaemia of the posterior ciliary vessels, causing significant optic nerve inflammation. Another postulated mechanism is immune-mediated demyelination. If not diagnosed early and treated aggressively, optic neuritis can result in irreversible visual loss [3,4].

Our report details a case of HZO-associated optic neuritis in an immunocompetent young adult, emphasising the importance of early neuroimaging, interdisciplinary involvement, and vigilant follow-up.

## Case presentation

A previously healthy 19-year-old female presented with complaints of pain, associated with swelling and multiple fluid-filled vesicular lesions over the right forehead, right upper eyelid, and extending to the tip of the nose for four days. She also had a low-grade fever for two days. She complained of diminution of vision in the right eye within five days of the onset of the rash. There was no history of trauma, similar symptoms in the past, or previous neurological illnesses. She had a childhood history of chickenpox at the age of seven, and she was uncertain about her immunisation history. On admission, she was alert and afebrile, with stable vitals. Examination of the right eye revealed upper lid swelling with crusted vesicles involving the V1 dermatome and a positive Hutchinson’s sign. Anterior segment examination showed conjunctival congestion and two dendritic epithelial erosions suggestive of herpetic keratitis. Visual acuity of the right eye was hand movements close to face (HMCF), with compromised colour vision, and a grade three relative afferent pupillary defect (RAPD) was observed. The right eye posterior segment appeared normal. The left eye had a visual acuity of 20/20, and the anterior segment and fundus were unremarkable.

**Figure 1:**
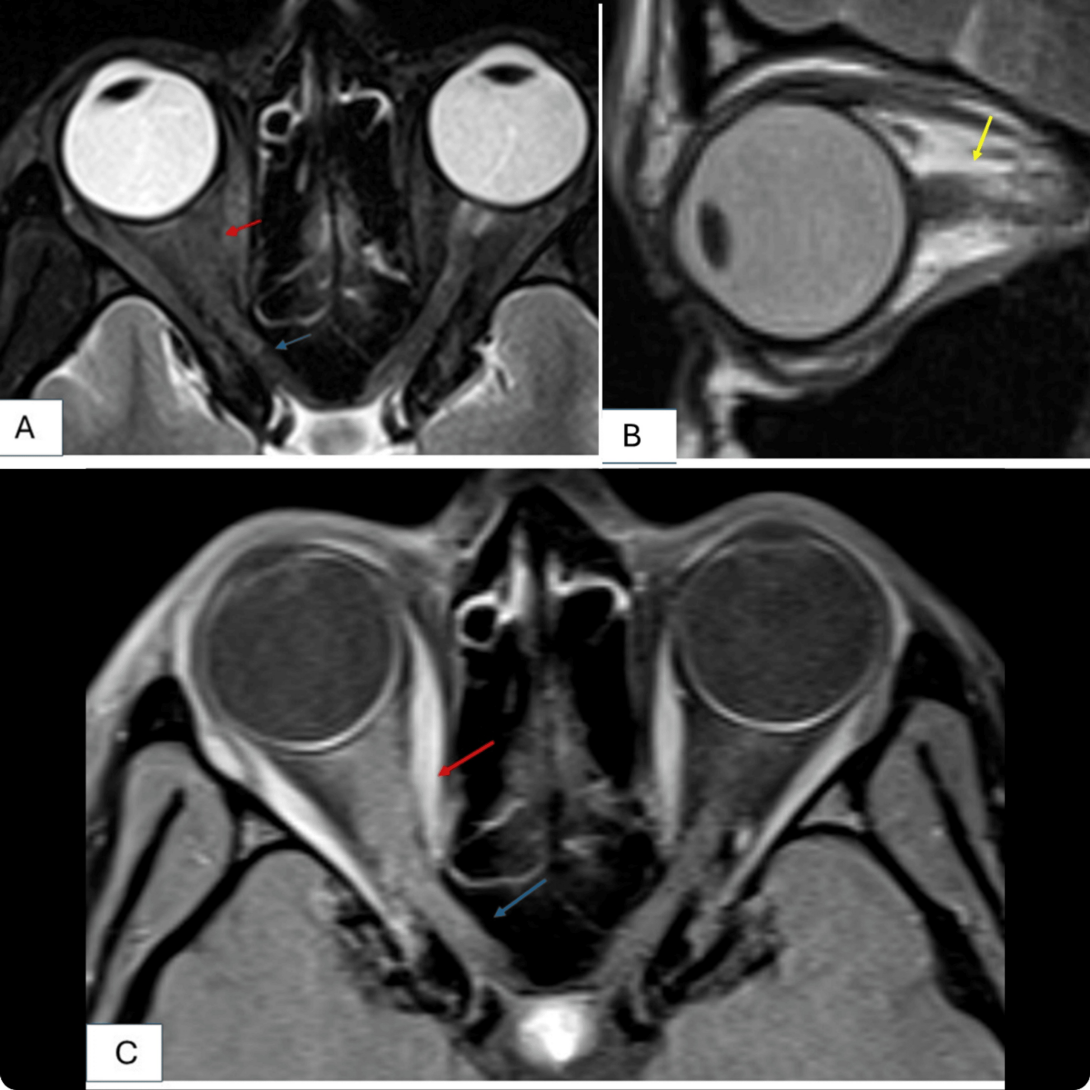
Neuroimaging A) Axial T2-weighted image of the MRI brain and orbit reveals a thickened intraconal (red arrow) and intracanalicular (blue arrow) part of the right optic nerve sheath complex. B) Sagittal T2-weighted image showing hyperintensities posterior to the globe, with surrounding fat stranding (yellow arrow). C) Axial T1-weighted post-contrast image showing homogeneous enhancement of the thickened intraconal (red arrow) and intracanalicular (blue arrow) part of the right optic nerve.

Laboratory investigations showed a haemoglobin level of 8.6 g/dL (12-16 g/dL), with a microcytic hypochromic picture suggestive of iron deficiency anaemia. The white blood cell count was 8340/mm³ (4000-11,000/mm³), and platelets were 2.49 lakh/mm³ (1.5-4.0 lakh/mm³). Liver and renal function tests, viral markers, thyroid function tests, and autoimmune markers were within normal limits. Chest X-ray and abdominal ultrasound were unremarkable. In suspicion of optic nerve involvement due to reduced visual acuity and RAPD, a contrast-enhanced magnetic resonance imaging (MRI) of the brain and orbits was performed. It revealed thickening of the intraconal and intracanalicular segments of the right optic nerve sheath complex, with perineural fat stranding and homogeneous post-contrast enhancement. These findings were consistent with right-sided optic neuritis (Figure 1). Visual evoked potential (VEP) revealed a delayed P-100 response on the right side, corroborating the above-mentioned diagnosis.

In view of the normal appearance of the right optic disc on fundus examination, a diagnosis of right HZO with retrobulbar optic neuritis was made. Neurologist and physician opinions were sought, and intravenous acyclovir (500 mg TDS) was initiated for seven days, followed by oral valacyclovir 1 g three times a day for six weeks. In view of MRI-confirmed optic neuritis, and under antiviral cover, intravenous methylprednisolone (IVMP) 1 g/day for five days was initiated, followed by a tapering regimen of oral prednisolone (1 mg/kg/day). Topical acyclovir eye ointment 3% five times a day for the right eye was also administered. During corticosteroid therapy, she developed significant hyperglycaemia, which was managed effectively with insulin, and later discontinued. She was started on oral iron supplementation for her anaemia, along with dietary modifications.

**Figure 2:**
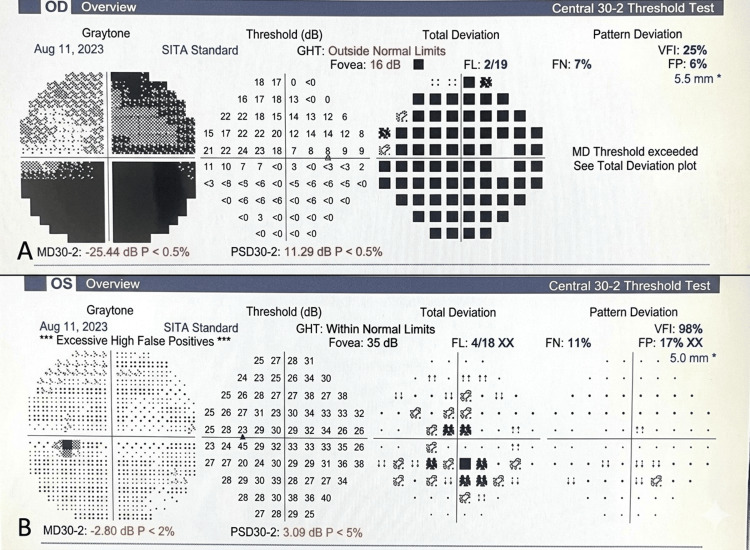
Perimetry A) Visual field chart of the right eye, demonstrating extensive loss of the peripheral field, with minimal sparing of central vision. The marked reduction of mean deviation (-25.44) signifies extensive global visual field impairment, which is an indicator of severe axonal involvement in herpes zoster ophthalmicus (HZO) optic neuritis. B) Normal visual field chart of the left eye.

On treatment completion, her right eye visual acuity improved from HMCF to 20/25. However, visual field testing, performed after visual recovery and resolution of the vesicular rash, showed generalised constriction of visual fields with central sparing in the right eye; the left eye was unremarkable (Figure 2). Global visual field impairment could indicate severe axonal loss in the setting of HZO optic neuritis, indicating poor visual prognosis.

**Figure 3:**
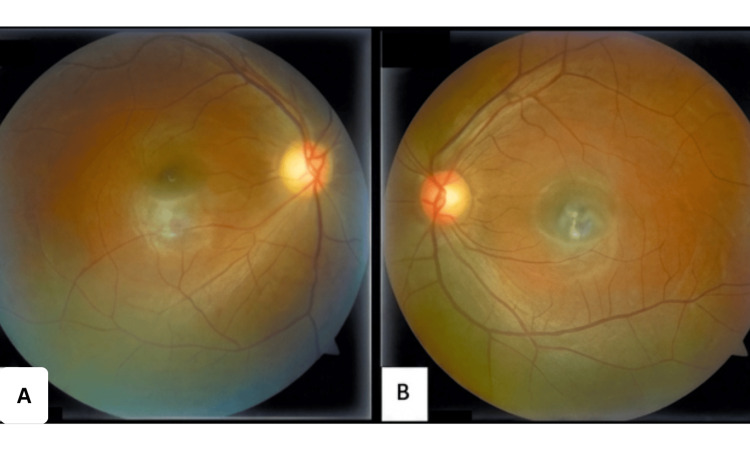
Fundus photograph Retinal imaging performed three months after initial presentation. A) The right fundus shows temporal pallor of the optic disc. B) The left fundus appears normal.

The corneal lesions had resolved, and there was no recurrence of active inflammation on serial follow-up. MRI repeated at six weeks showed regression of optic nerve sheath enhancement, suggesting resolution of active inflammation. Her haemoglobin improved to 10.4 g/dL after iron therapy, and blood glucose levels normalised. At the three-month follow-up, fundus examination of the right eye revealed temporal disc pallor (Figure 3).

## Discussion

HZO is a reactivation of latent VZV in the trigeminal ganglion, and its ophthalmic involvement can range from superficial keratitis to vision-threatening posterior segment and neuro-ophthalmic complications [1,2]. The risk of ocular complications is increased when Hutchinson’s sign is present, due to involvement of the nasociliary branch supplying the cornea and iris [4]. Optic neuritis as a complication of HZO is rare and poses diagnostic and therapeutic challenges. The underlying mechanisms for HZO optic neuritis proposed include direct viral invasion leading to necrotising inflammation, immune-mediated demyelination, and ischaemic injury inducing vasculitis [5-8]. The diagnosis may be delayed, especially if the patient has severe lid and corneal lesions, which mask the aetiology for diminution of vision. The use of cycloplegic eye drops to prevent ciliary spasm may further compromise the identification of pupillary reactions. Fundus examination is usually normal in the early stage. Neuroimaging is crucial for the diagnosis of optic nerve inflammation. VEP has an adjuvant role in the confirmation of anterior conduction pathway deficit [9]. In our patient, partial recovery of visual acuity was achieved; however, temporal pallor of the right optic disc, corresponding with a global reduction of visual field, indicates severe axonal loss of the right optic nerve.

Several cases of HZO optic neuritis have been described in the literature, as elucidated in Tables 1-2.

**Table 1:** Literature review of HZO optic neuritis case reports HZO: herpes zoster ophthalmicus; IOP: intraocular pressure; IVMP: intravenous methylprednisolone; HMCF: hand movements close to face

Author	Patient Demographic (Age/Sex)	Visual Acuity (Affected Eye)	Ocular Features	Treatment and Outcome
Al-Sadi et al. [1]	Late 40s/male	Not assessed due to lid oedema	Right abducens nerve palsy, dendritic corneal ulcer and elevated IOP. MRI suggestive of optic neuritis	IV acyclovir followed by oral valacyclovir, topical prednisolone acetate eyedrops and beta blockers; lost to follow-up
Ryu et al. [3]	Elderly male	20/20 right eye, no light perception left eye	Left eye abducens nerve palsy with optic neuritis and retinal vasculitis; right eye inferonasal field defect	IVMP 1 gm for three days followed by oral steroids and IV acyclovir; stabilisation in the right eye, no visual improvement in the left eye
Matsuo and Iguchi [4]	Elderly male	20/60	Left eye abducens nerve palsy with bilateral optic neuritis	Observation; improvement of visual acuity to normal and reduction of optic disc swelling
Phang et al. [10]	Late 20s/male	HMCF	Left eye retrobulbar optic neuritis with normal fundus	IV acyclovir followed by oral acyclovir; no improvement in vision for one year
Afshar et al. [11]	Early 60s/male	20/70	Right eye abducens nerve palsy with retrobulbar optic neuritis	Oral valacyclovir and corticosteroids; improved to 20/25 after one month, gaze palsy improved by six months
Hong and Yang [9]	Child/female	20/50	Right eye HZO and optic neuritis	Combined antiviral and steroid therapy is effective; good recovery

**Table 2:** Summary of relevant literature on HZO optic neuritis treatment outcomes HZO: herpes zoster ophthalmicus

Author	Visual Acuity	Demographics	Treatment Outcomes	Conclusion
Hong et al. [11]	0.4 (right eye)	Child with HZO and optic neuritis	Improved to 0.8 at one month with acyclovir + steroids	Combined antiviral and steroid therapy effective; good recovery possible
Pourmahdi-Boroujeni et al. [12]	Variable; often moderate to severe vision loss	Adults with HZO-related optic neuritis, eye pain and blurred vision	52.8% mild/no impairment; 36.3% clinical blindness	Early diagnosis and combined corticosteroid + antiviral therapy improve prognosis
Shahriari et al. [13]	Not always detailed; vision loss is common	Ophthalmoplegic HZO patients	Corticosteroids showed better improvement than antivirals alone	Corticosteroids enhance recovery when combined with antivirals
Sverdlichenko et al. [14]	Variable acuity; some severe cases	HZO with cranial nerve palsies	75% recovery with combination therapy; 85.7% with antivirals alone	Combined therapy is beneficial in many patients
Yuan et al. [15]	Not explicitly reported	HZO optic neuropathy cases	65% improvement with corticosteroids; some improvement with antivirals alone	Visual recovery possible; the role of IV vs oral antivirals needs clarification

In our literature review, the combined use of intravenous corticosteroids and antiviral agents in the treatment of optic neuropathy secondary to HZO was found to be beneficial, as it helped to reduce inflammation and viral replication [12-15]. This could potentially help in restoring visual acuity. However, the evidence underscores the necessity to balance these benefits with caution, due to variability in patient responses and a risk of retinal spread resulting in retinal necrosis. Intravenous antivirals such as acyclovir remain the cornerstone of treatment for HZO-related optic neuropathy, with robust guidelines supporting initiation within 72 hours of symptom onset to reduce viral load and prevent progression. Acyclovir and its prodrug valacyclovir are effective against VZV and are recommended for a minimum of 7-10 days in acute HZO [12,16]. Extended antiviral therapy may be considered in patients with delayed presentations or complications such as optic neuritis. Our patient received six weeks of antiviral therapy due to persistent symptoms.

The adjunctive use of systemic corticosteroids aims to control the intense inflammatory response triggered by viral infection and immune-mediated optic nerve damage. Corticosteroids may reduce optic nerve oedema and demyelination, potentially preserving visual function. Several case series and meta-analyses report improved or stabilised vision with combined corticosteroid and antiviral therapy [13,14]. High-dose and pulse corticosteroid regimens have been utilised safely, with close monitoring, demonstrating favourable outcomes in a significant subset of patients.

Nonetheless, the use of corticosteroids warrants careful clinical judgment. Risks include aggravating viral replication if antivirals are insufficient, worsening immunosuppression, especially in diabetics or immunocompromised patients, and masking secondary infections. Some literature cautions against steroids in the absence of adequate antiviral coverage or in patients with contraindications to immunosuppression [9,17]. Visual prognosis remains highly variable despite treatment, with some patients suffering permanent vision loss, likely due to ischaemic optic nerve damage or late presentation [12,17].

In conclusion, the current evidence supports a therapeutic strategy that prioritises prompt initiation of intravenous antivirals combined with cautiously administered systemic corticosteroids to maximise the chances of visual recovery in HZO-related optic neuropathy [8]. Early diagnosis and treatment initiation are critical. Individual patient factors and risk profiles must be carefully assessed to tailor therapy, mitigate adverse effects, and optimise outcomes. Our case demonstrates that systemic steroids, when combined with antiviral coverage, can be used safely, albeit with close metabolic monitoring. Steroid-induced hyperglycaemia and underlying iron deficiency anaemia, both of which can negatively impact neurological recovery, were managed promptly in this case. These comorbid conditions highlight the need for multidisciplinary involvement in managing such complex patients [18].

## Conclusions

Optic neuritis is a rare but serious complication of HZO that may occur even in immunocompetent individuals. Documentation of pupillary reaction and early neuroimaging is essential in patients with vision loss following HZO to confirm optic nerve involvement. Timely and optimal treatment with antivirals and corticosteroids can yield favourable outcomes with partial visual recovery. Multidisciplinary care, including neurology, ophthalmology, and endocrinology, is key to managing both the primary condition and treatment-related side effects.
